# Post-transcriptional regulation of Rad51c by miR-222 contributes cellular transformation

**DOI:** 10.1371/journal.pone.0221681

**Published:** 2020-01-10

**Authors:** Emilio Rojas, Monica Martinez-Pacheco, Maria Alexandra Rodriguez-Sastre, Paulina Ramos-Espinosa, Mahara Valverde

**Affiliations:** 1 Universidad Nacional Autónoma de México, Instituto de Investigaciones Biomédicas, Departamento de Medicina Genómica y Toxicología Ambiental, Mexico City, C.U., México; 2 Center for Genomic Sciences, UNAM, Cuernavaca, Mexico; University of Kansas School of Medicine, UNITED STATES

## Abstract

DNA repair inhibition has been described as an essential event leading to the initiation of carcinogenesis. In a previous study, we observed that the exposure to metal mixture induces changes in the miR-nome of the cells that was correlated with the sub-expression of mRNA involved in processes and diseases associated with metal exposure. From this analysis, one of the miRNAs that shows changes in its expression is miR-222, which is overexpressed in various cancers associated with exposure to metals. *In silico* studies showed that a possible target for the microRNA-222 could be Rad 51c, a gene involved in the double-stranded DNA repair. We could appreciate that up-regulation of miR-222 reduces the expression both gene and as a protein expression of Rad51c by RT-PCR and immunoblot, respectively. A luciferase assay was performed to validate Rad51c as miR-222 target. Neutral comet assay was performed in order to evaluate DNA double-strand breaks under experimental conditions. Here, we demonstrate that miR-222 up-regulation, directly regulates Rad51c expression negatively, and impairs homologous recombination of double-strand break DNA repair during the initiation stage of cell transformation. This inhibition triggers morphological transformation in a two-stage Balb/c 3T3 cell assay, suggesting that this small RNA acts as an initiator of the carcinogenesis process.

## Introduction

The understanding of cancer has evolved dramatically during the last decades with the knowledge that cancer cells acquire their characteristics at different times during the development of cancer, in various microenvironments, through various mechanisms [1,2]. Genome instability is defined as an increased tendency of the genome to acquire genetic alterations [3]. It occurs when several processes involved in the maintenance and replication of the genome are dysfunctional or when there is an increasing exposure to carcinogens. The instability of the genome is an enabling feature that is causally associated with the acquisition of the distinctive characteristics of cancer. Then, tumor progression is the result of the continuous selection of variant subpopulations of malignant cells that have acquired increasing levels of genetic instability [4].

The instability of the genome is associated with cellular deficiency in the response to DNA damage. To preserve genomic integrity, cells have developed a complex cellular system to detect and repair DNA damage. Double-stranded DNA breaks (DSB) are one of the most severe types of DNA damage and are repaired by error-free homologous recombination (HR) or non-homologous end-joining (NHEJ). Other types of DNA damage, such as errors that occur during replication, base oxidation, or the formation of covalent bonds between bases, are processed by mismatch repair (MMR), base excision repair (BER) and nucleotide excision repair (NER) respectively. The mechanisms of DNA repair allow the maintenance of the integrity of genetic information. Hereditary and somatic defects in the genes involved in these mechanisms could lead to genome instability and favor the development of various human cancers. For example, mutations in NER genes represent a very important factor in the susceptibility to developing skin cancer [5], and mutations in HR genes predispose to various cancers, including cancer of the skin, ovary, breast, lymphomas and leukemia [6]. Nevertheless, studies of next generation sequencing realized in the last years have revealed that the instability of the genome, in the majority of the sporadic human cancers, is not due to mutations in genes associated to these routes [7], which raises the need to consider that there is an aberrant post-transcriptional regulation.

The regulation of gene expression at the posttranscriptional level can occur through short sequences of non-coding RNA of approximately 21 nucleotides known as microRNAs (miRNAs). The miRNAs are able to bind messenger RNAs and inhibit their translation [8,9] and their interaction is mediated by partial sequence homology. In spite of their relatively small number, computational and experimental studies have indicated that miRNAs can control the expression of most, if not all, human protein-coding genes [10–12]. Hence, miRNAs function is indissolubly linked to their targets and, because each miRNA can bind and modulate hundreds of targets, any cell function is in fact regulated by miRNAs. miRNAs are key regulators of numerous biological functions. Deviation from their normal expression has been involved in human diseases [13–18].

In this context studies showed that miRNAs are involved in the regulation of DNA repair through the modulation of BER, MMR, NER, NHEJ and HR mechanisms (Table 1) [19–34].

**Table 1:** Negative regulation of DNA repair gene expression through miRNAs.

microRNA	DNA repair Gene	Reference
miR-421	ATM ATR	22
miR-16 miR-34c miR-199	UNG	23
miR-373	RAD 23b	24
miR-192	ERCC2 ERCC3	25
miR-31-5p miR-155	Mlh1 Msh6	26 27
miR-21	Msh2	28 29
miR-210	Rad 52	24
miR-96 miR-103 miR-107 miR-155	Rad 51a Rad-51c	29 30 31
miR-9 miR-182 miR-1245	Brca1 Brca2	32 33
miR-7	Xrcc2	34

Among them, HR which recognized DNA double strand breaks (DSBs), is of our great interest due to its ability to maintain genomic stability and its important role in the development of diverse types of cancer [35]. Particular attention is paid to the Rad51 recombinase protein family implicated in this DNA repair mechanism and more specifically to the Rad51c member in light of the experimental data demonstrating its mutation or inactivation as a susceptibility factor of various types of cancer, including head and neck, breast, ovarian, and colorectal [35–38].

In a previous work we report that the mixture of metals of arsenic, cadmium and lead produces changes in the expression patterns of miRNAs which are correlated with changes in mRNA patterns, such a relationship could explain the effects of metals on cellular mechanisms included DNA repair [39]. One of the microRNAs that was observed to be overexpressed was the miR-222.

miR-222 is known to be up-regulated in established cancers [40–43] which have been related to these metals [44]. In light of this fact and given the previous studies of miR-222, the aim of the present study is to determine the role of miR-222 as an initiator stimulus of cellular transformation through the modulation of Rad51c protein expression in the two-stage Balb/c 3T3 cell assay.

## Materials and methods

### *In silico* identification of miR-222 target and Rad51c interaction

Prediction of complementary binding sites between the mature mmu-miR-222 (seed sequence: 5´-aGCUACAU-3´) and the 3´UTR of Rad51c mRNA (RefSeq ID: NM_053269) sequences was made using the miRWalk [45], microRNA [46] and miRbase [47] algorithms.

### Cell lines

The Balb/c 3T3 clone A31-1-1 is a *Mus musculus* (mouse) non-tumorigenic and non-immunosuppressed embryo fibroblast widely used for carcinogenicity test purposes. The strain was acquired on 2009 from the ATCC (American Type Culture Collection; VA, USA) and it has been cryopreserved in freeze medium (complete growth medium supplemented with 5% (v/v) DMSO (dimethyl sulfoxide)) on liquid nitrogen. All of the experiments were carried out using an early passage number of the subculture (passage 4) and employing a positive (known initiator and promoter) and negative (basal conditions culture) control for cellular transformation assays.

The Vero strain is an adult *Cercopithecus aethiops* (African green monkey) kidney cell vastly used for plasmid transfection that has been acquired from the same supplier and cryopreserved under the same conditions. All of the experiments were performed using an early passage number of the subculture (passage 5).

### Two-stage Balb/c 3T3 cell assay

**Fig 1:**
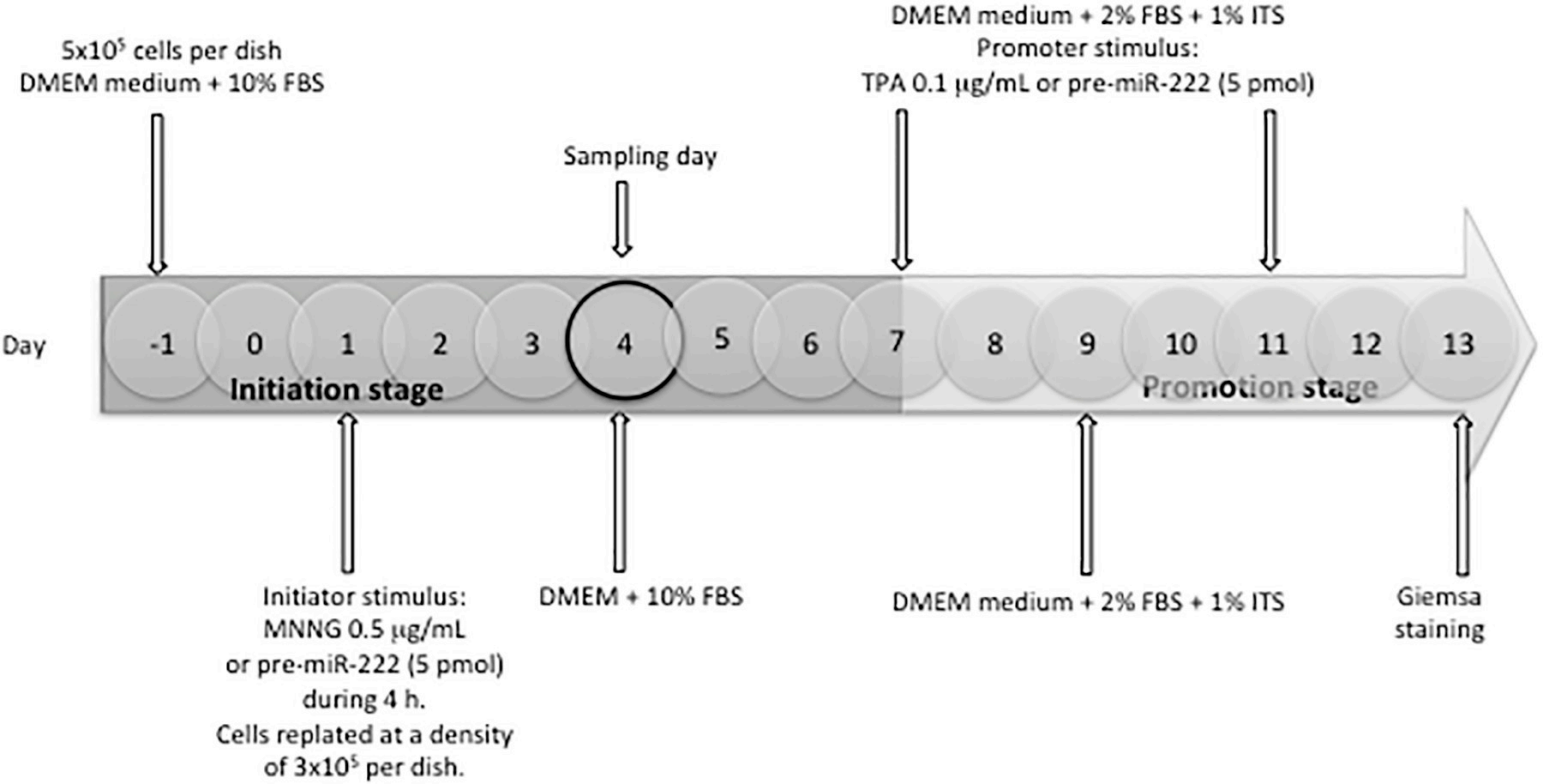
Scheme of the two-stage Balb/c 3T3 cell assay for miR-222 as initiator and/or promoter test. Pre-miR-222: 5 pmol; MNNG: positive initiator; TPA: positive promoter. Initiation stage (day 1–7), promotion stage (day 7–13).

The two-stage Balb/c 3T3 cell assay was performed as described previously with slight modifications [48,49]. The transformation protocol consisted of 13 days, divided in two phases: initiation between days 1 to 7 and promotion between days 7 to 13. Cells were plated at a density of 5x10^5^ cells per 100-mm dish in DMEM (Dulbecco’s modified eagle medium) supplemented with 10% FBS (fetal bovine serum). On day 1 of the assay, sub-confluent cells were exposed to the initiator stimuli during 4 h; MNNG (N-methyl-N-nitro-N-nitroso-guanidine) (0.5 μg/mL) as a positive control; pre-miR 222 (5 pmol), as experimental condition and untreated cells were used as a negative control. After such initiator treatment, cells were harvested and replated at a density of 3 x10^5^/dish. After 72 h (day 4), cultures were replenished with fresh DMEM medium supplemented with 10% FBS. On days 7 and 11, cells were replenished with medium supplemented with 1% ITS-A (insulin transferrin selenium-A) and 2% FBS, and pre-miR 222 (5 pmol) or TPA (12-O-tetradecanoylphorbol-13-acetate) (0.1 μg/ml) (positive control) was added as the promoter stimuli (Fig 1). At day 9, cultures were replenished with fresh medium without promoter stimuli (Fig 1). On day 4 (during the initiation stage) cells were harvested for the experimental procedures (Fig 1). On days 13, cells were fixed with ethanol, stained with 10% aqueous giemsa and scored for foci formation (Fig 1). Transformed foci were scored according to criteria that discriminate transformed foci on the basis of 3 morphological characteristics: (1) basophilic staining; (2) a dense layer formation; and (3) random orientation of cells at the edges of foci [49]. Foci less than 2-mm diameter were not scored. Relative Colony Formation Efficiency (RCFE) was calculated as the number of foci per dish in experimental conditions/number of foci per dish in control conditions. The experimental conditions were as follows: control (untreated cells); pre-miR-222/TPA to probe the initiator capacity of miR-222; MNNG/Pre-miR-222 to probe the promoter capacity of miR-222; and pre-miR-222/pre-miR-222 to probe both capacities of miR-222 (Fig 1).

### Cell viability assay

Cell viability was measured by the dual stain FDA/EtBr method [50]. FDA is taken up by cells which through esterases activity transform the non-fluorescent FDA into the green fluorescent metabolite. Meanwhile, nuclei of death cells are ethidium bromide stained and visualized as red fluorescence. Cells were then analyzed under a fluorescence microscope (Olympus BMX-60 with a UM61002 filter). One hundred randomly chosen cells per condition were evaluated and the results are expressed as percentages.

### RNA isolation

On day 4 of the Balb/c 3T3 transformation assay, during the initiation stage, control and transfected cells were harvested with 0.2% PBS–EDTA (phosphate buffered saline-ethylenediaminetetraacetic acid) and centrifuged to remove the medium. Total RNA was immediately extracted from 2 × 10^5^ cells using the ZR RNA MicroPrep (Zymo Research; CA, USA) isolation kit according to the manufacturer’s protocol for miRNA analysis and with the Maxwell 16 LEV Simply RNA cells kit (Promega; WI, USA) on a Maxwell 16 Instrument (Promega) for mRNA evaluation. Total RNA was analyzed on a Nanodrop 1000 (Thermo Fisher Scientific; DE, USA) spectrophotometer for mRNA integrity evaluation and sample quantification; samples were then aliquot and stored at −80°C.

### miR-222 RT-qPCR

*Mus musculus* miR-222 expression was determined by RT-qPCR (reverse transcription-quantitative polymerase chain reaction), and all of the reagents were purchased from the same supplier (Applied Biosystems; CA, USA). For miRNA cDNA (complementary DNA) synthesis, RNA was reverse transcribed with the TaqMan MicroRNA Reverse Transcription kit and TaqMan MicroRNA Assay hsa-miR-222 primer, and real time PCR was performed using TaqMan Universal Master Mix II (no UNG (uracil-N-glycosylase)) along with labeled TaqMan MicroRNA Assay hsa-miR-222 primers. The mammalian U6 non-coding small nuclear RNA (U6 snRNA) was used for data normalization, and the 2^-ΔΔCT^ comparative method (described on the Applied Biosystems ABI Prism User Bulletin No. 2 and explained by Livak and Schmittgen [51]) was applied to calculate relative changes in gene expression determined from quantitative experiments.

### Rad51c mRNA RT-PCR

Rad51c gene expression was assessed by endpoint RT-PCR using the Access RT-PCR System kit (Promega) and mouse Rad51c recombinase primers (IDT; IA, USA) in cells in which miR-222 expression was up-regulated; we employed the mouse Hprt1 gene as an endogenous control across all experiments. The RT-PCR products were resolved on a 2.5% agarose gel containing EtBr (0.5 mg/mL), visualized under UV (ultraviolet) light on the MiniBIS Pro Imaging System (DNR; JRS, Israel) and quantified by means of band intensity with the Kodak 1D Image Analysis v3.5 software (Kodak; NY, USA).

### Protein extraction and Rad51c immunoblot

Whole cell protein was extracted with Radio Immunoblot Precipitation Assay buffer (RIPA) and conventionally treated for specific immunodetection by western blot technique [52] of mouse Rad51c protein in cells treated with miR-222 or transfection control. We used an anti-Rad51c polyclonal antibody that recognizes RELVGYPLSPAVRGKGKLVAAGFQTAED, corresponding to N terminal amino acids 3–28 of Mouse Rad51C (Cat. ab95201, Abcam; Cambs., UK) and detected ß-tubulin protein as an endogenous control for the experiments with a mouse anti-ß-tubulin monoclonal antibody (Cat. 322600, Invitrogen, Camarillo, CA, USA). Horseradish Peroxidase (HRP)-coupled goat anti-rabbit IgG (immunoglobulin G) monoclonal (Cat. 816129, Invitrogen, Camarillo, CA, USA) and goat anti-mouse IgG monoclonal (Cat. 626520, Invitrogen, Camarillo, CA, USA) secondary antibodies were utilized for Rad51c and ß-tubulin radiographical detection, respectively, with the Immobilon Western Chemiluminescent HRP Substrate kit (Millipore; MA, USA). Protein quantification was performed with Kodak 1D Image Analysis v3.5 software and expressed as band intensity at optical densities (D.O.).

### miR-222 and Rad51c luciferase assay

A luciferase reporter assay was performed in the easy-to-transfect Vero (African green monkey kidney cells) cell line to test the regulatory effect of miR-222 over Rad51c mRNA. The pEZXMT05-Rad51c-3´UTR plasmid containing the Rad51c 3´UTR sequence, obtained from a public domain gene sequence database, was inserted downstream of the secreted *Gaussia luciferase* (GLuc) reporter gene inside a vector system driven by the SV40 promoter for expression in mammalian cells. The vector was purchased from *GeneCopoeia* (MD, USA), and the cells were transfected with EndoFectin PLUS reagent from the same supplier. After successful transfection, a mRNA consisting of the GLuc and the 3' UTR target sequence is transcribed; thus, when it is co-transfected with the synthetic mmu-miR-222 mature sequence (5 pmol) (GeneCopoeia, MD, USA), the study of mRNA-miRNA target interaction can be easily observed in terms of the Gluc activity detected in the culture medium, which was measured using the Secrete Pair Dual Luminescence Assay kit (GeneCopoeia, MD, USA). As an internal control, a Secreted Alkaline Phosphatase (SEAP) activity reporter, driven by a CMV promoter, was cloned into the same vector and used for transfection-normalization across sample comparison. Additionally, a negative control plasmid lacking the Rad51c 3´UTR sequence (pEZXMT05) was used to demonstrate the null effect of miR-222 on GLuc assay when Rad51c is not present. GLuc and SEAP activities were determined on the FLx800 microplate fluorescence/luminescence Reader (BioTek Instruments; VT, USA) and analyzed with the KCjunior v1.41.8 software (BioTek, VT, USA).

### miR-222 precursor molecule transfection

Balb/c 3T3 cells were transfected with 5 pmol of the miR-222 precursor molecule (pre-miR-222) for 24 h using the siPORT NeoFX transfection solution (all reagents from Ambion; MA, USA) to induce the up-regulation of miR-222 expression, which was confirmed by RT-qPCR as described earlier in this manuscript.

### Ri-1 inhibition of RAD51

Balb / c 3T3 cells were treated with 10 μM of RI-1 for 24 hours, after which time the cells were treated with Doxorubicin (50 μM) for one hour. After 24 hours of recovery under optimal conditions, DNA damage and phosphorylation of H2AX histone and ATM was quantified.

### DNA-DSB determined by neutral comet assay

To evaluate DNA DSBs induced by the up-regulation of miR-222 expression, the neutral comet assay was performed in Balb/c 3T3 cells as described previously [53] under pre-miR-222 transfection conditions. Slides were prepared per duplicate; 50,000 cells were mixed with 75 μL of 0.7% LMP (low melting point) agarose solution and loaded onto microscope slides prelayered with 150 μL of 0.5% normal melting point agarose, after which a third layer of LMP agarose was added. After incubation with lysis buffer (cold EDTA sodium salt 30 mM and SDS 0.5% pH 7) for 24 hours, the slides were subjected to unwind for 2 h and electrophoresis at 25V, 20mA for 25 min in buffer (boric acid 90 mM, EDTA 200 mM and Tris base 117 mM pH 7.8). Slides were dehydrated with 96% ethanol, stained with EtBr and visualized under a fluorescence microscope (20x) to determinate the Olive Tail Moment (OTM) of 100 comets/slide with the Komet 5 software (Komet Imaging, Ltd. UK). 3-Gy gamma radiation was applied as DSB-DNA inductor to challenge DSB repair mechanism [54].

### Statistical analysis

All of the data were analyzed using a Welch-corrected unpaired two-tailed *t*-test to determine differences between experimental conditions using the Prism 6 (GraphPad; CA, USA) statistics package. Results with a *p-value* < 0.05 were considered statistically significant. A One-way ANOVA was performed for determine transformation capacity, and Tukey post-hoc (95% CI (confidence interval)) test was applied for comparisons between groups. Results with a *p*-values <0.05 were considered as statistically significant.

## Results

### Basal values of miR-222 and Rad 51c in Balb/c 3T3 cells

To determine Rad 51c regulation by miR-222 we began with determination of the basal expression levels of miR-222, in addition, we evaluated expression changes of miR-222 across cell transformation process (the results can be seen in Table 2). With respect to the basal values of RAD51c we found that the genetic expression with reference to the HPRT gene (reference gene) is 0.615± 0.15 and the protein expression with respect to Tubulin (reference protein) is 1.57 ± 0.33.

**Table 2:** miRNA expression across the cell transformation assay. Basal value of miR-222 were calculated with respect to the value of U6 (reference control). Fold change values of Pre-miR-22 and Anti-miR-222 were calculated with respect to control values.

	4h	8h	24h	48h	72h	96h	336h
miR-222/U6	2.44 ± 0.03	2.34 ± 0.08	2.40± 0.09	2.29 ± 0.06	3.5 ± 0.09	1.47 ± 0.59	nd
Pre-miR-222/Control	16.03 ± 1.5	50.09 ± 3.2	44.07 ± 4.5	18.22 ± 1.8	130.2 ± 2.2	39.21 ± 0.44	0.38 ± 0.1
Anti-miR-222/Control	-7.64 ±0.3	-9.96±0.4	-5.79 ±0.2	-2.70 ± 0.1	-3.85 ±0.1	nd	nd

### Prediction of miR-222 and Rad51c interaction

We identified the possible direct binding of the miR-222 seed sequence on five different sites throughout the 3`UTR of Rad51c mRNA using the miRWalk, microRNA and miRBase databases. The miRWalk algorithm allowed us to predict the regulation of this gene target with a *p-value* < 0.05 (p = 0.0413), strengthening the possibility of its negative regulation by miR-222.

### Rad51c is a direct target of miR-222

**Fig 2:**
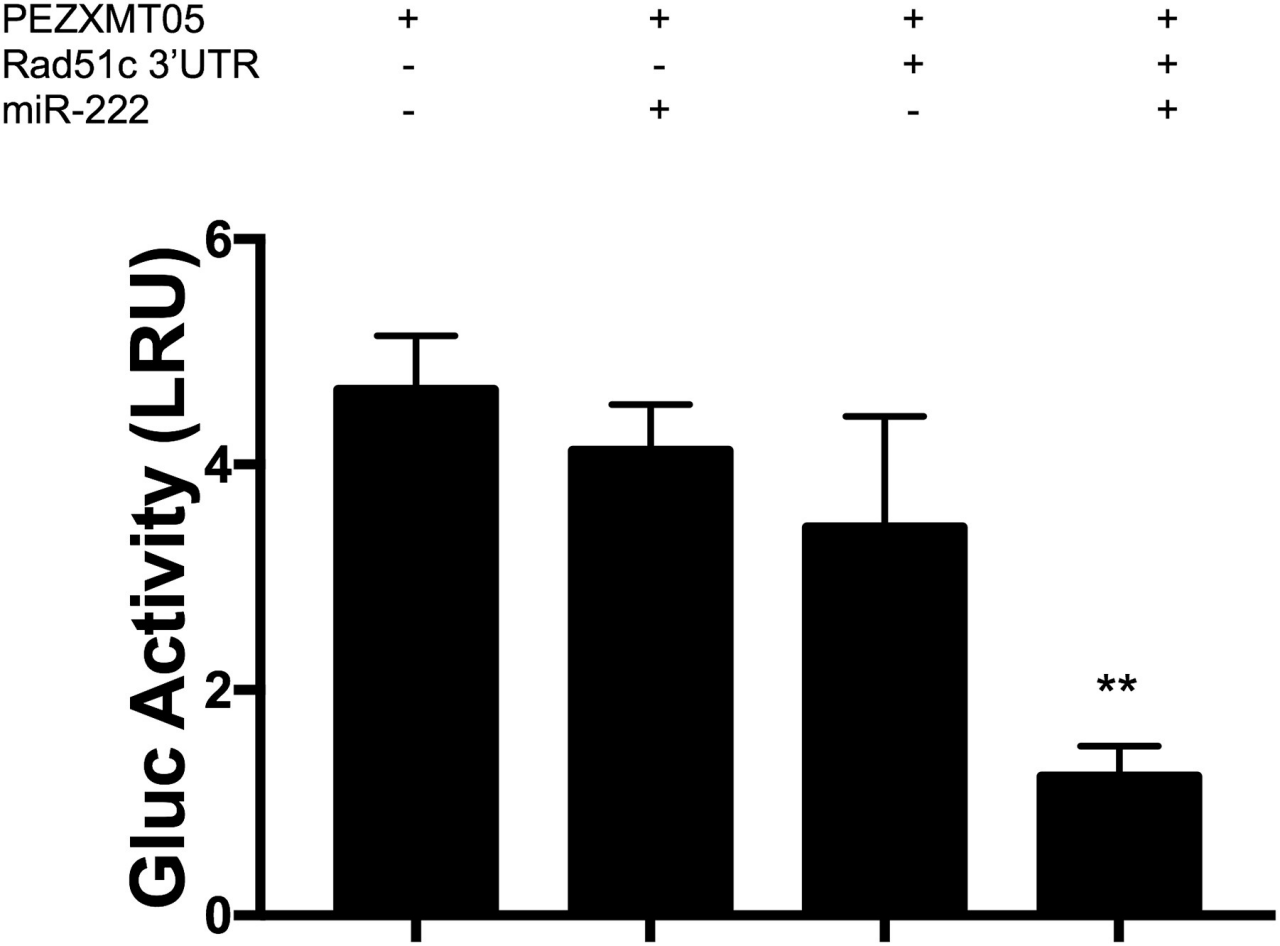
*Gaussia Luciferase* (GLuc) activity in Vero cells transfected with a plasmid containing the 3´UTR of Rad51c downstream the GLuc gene (pEZXMT05-Rad51c-3´UTR) co-transfected with or without the mature sequence mimic of miR-222; the results are expressed as Luminescence Relative Units (LRU). Endogenous control: Alkaline Phosphatase (AP), pEZXMT05: negative control, N = 3, mean±±SE two-tailed unpaired t-test, *p<0.05, ** p< 0.01.

We tested this hypothesis, (direct negative regulation of Rad51c by miR-222) by transfecting the Vero strain with a luciferase reporter plasmid containing the 3´UTR of the gene in question. The pEZXMT05-Rad51c-3´UTR plasmid was co-transfected with the synthetic mmu-miR-222 mature sequence, and a plasmid lacking the Rad51c 3´UTR sequence (pEZXMT05) was used as negative control. Our results revealed an approximately 80% decrease in GLuc activity when the cells were transfected with miR-222 but no significance differences when the pEZXMT05 plasmid was co-transfected with the miRNA sequence (Fig 2).

### Precursor-induced miR-222 over-expression inhibits both gene and protein Rad51c expression

Transfecting Balb/c 3T3 cells with pre-miR-222 for 24 h induced up-regulation of miR-222. The cellular viability assay demonstrated that the transfection of the Pre-miR-222 molecule had no cytotoxic effect (92.22%±0.88 viability) compared to the siPORT (transfection reagent) condition (89.66%±1.69) (Table 3).

**Table 3:** Viability and miR-222 expression. Percentage of cellular viability, measured as metabolic activity using the FDA/EtBr method, of Balb/c 3T3 cells. Relative expression of mmu-miR-222 in Balb/c 3T3 cells. The results of RTqPCR are represented in terms of 2^- ΔΔCT^; endogenous control: snRNA U6. Both determinations were realized in day 4 during initiation stage of transformation assay. N = 3, mean±SE two-tailed unpaired t-test, *p<0.05.

	Transfected cells with miR-222 precursor	
	Viability %	miR-222 expression
**Control**	95 ± 0.5	1.0 ± 0.10
**Siport**	89.66 ± 1.7	1.00 ± 0.09
**Pre-miR-222**	92.22 ± 0.9	39.21 ± 0.44*

**Fig 3:**
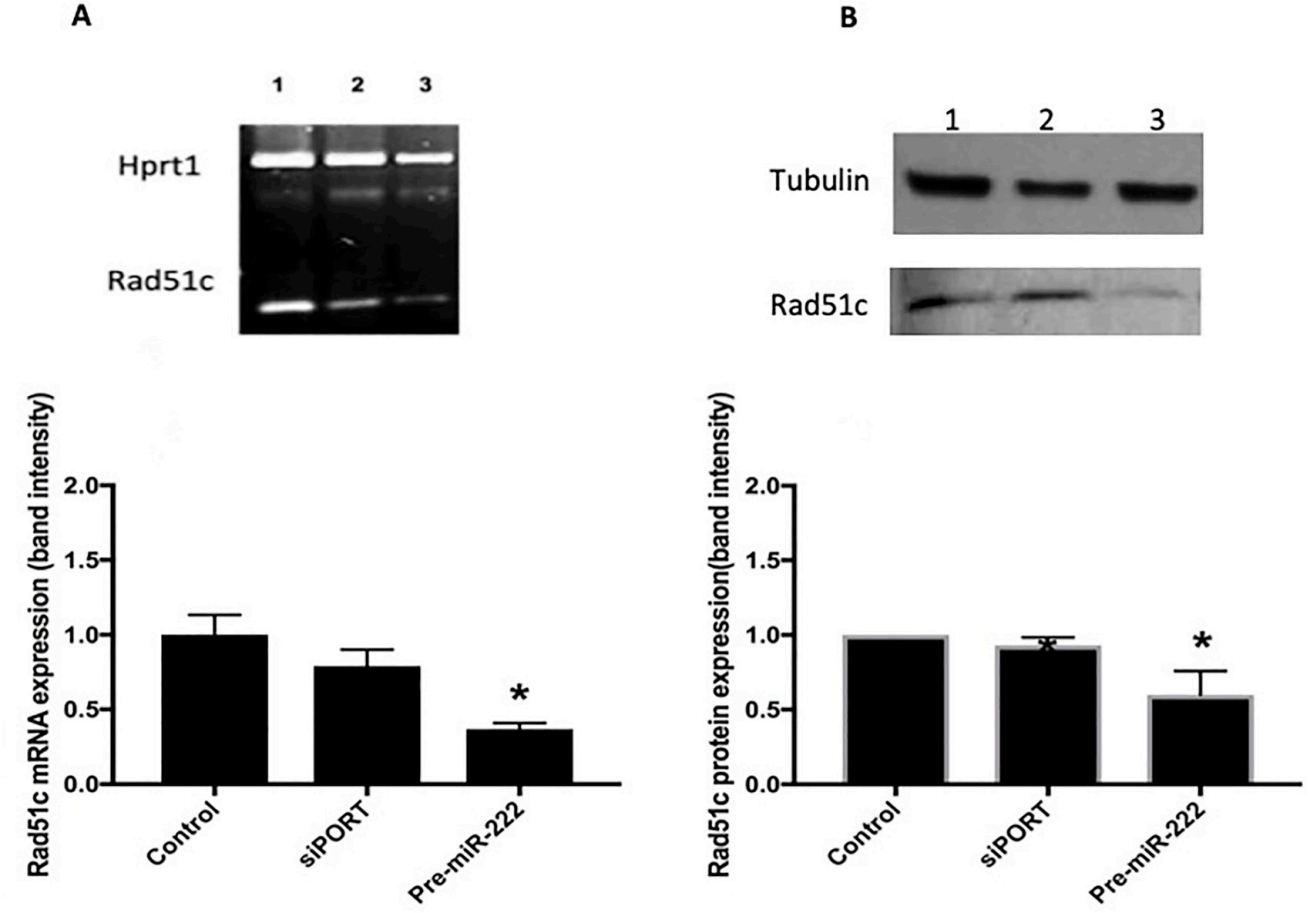
Rad51c expression changes on day 4 (initiation phase) of the transformation assay. **(A)** Relative gene expression of Rad51c assessed by RT-PCR in Balb/c 3T3 cells on day 4 (initiation phase) of the transformation assay. 1.- Control cells, 2 Cells transfected with Siport, 3 Cells transfected with the precursor molecules of miR-222 (pre-miR-222). Band intensity was normalized against endogenous control: Hprt1; N = 3, mean±SE., two-tailed unpaired t-test, *p<0.05. **(B)** Relative Rad51c protein expression measured by immunoblot in Balb/c 3T3 cells on day 4 (initiation stage) of the Two stage Balb/c 3T3 cell assay. 1.- Control cells, 2.- Cells transfected with Siport, 3.- Cells transfected with the precursor molecules of miR-222(pre-miR-222). Band intensity was normalized against endogenous control: α-tubulin. N = 3, mean±SE, two-tailed unpaired t-test, *p<0.05.

Seventy-two hour after transfection, on day 4, we evaluated the expression of miR-222 by RT-qPCR and observed a significant elevation in the levels of this miRNA (2^- ΔΔCT^ = 39.21±0.44) relative to the siPORT condition (2^- ΔΔCT^ = 1.0±0.09) (Table 3). Cells over-expressing miR-222 exhibited a decrease in Rad51c mRNA of approximately 50% (0.37±0.04) compared with the siPORT condition (0.79±0.11) (Fig 3A) as well as a significant decrease, of around 40%, in the Rad51c protein expression (0.60±0.1) relative to the siPORT condition (0.93±0.05) (Fig 3B).

### DNA DSBs in cells over-expressing miR-222

**Fig 4:**
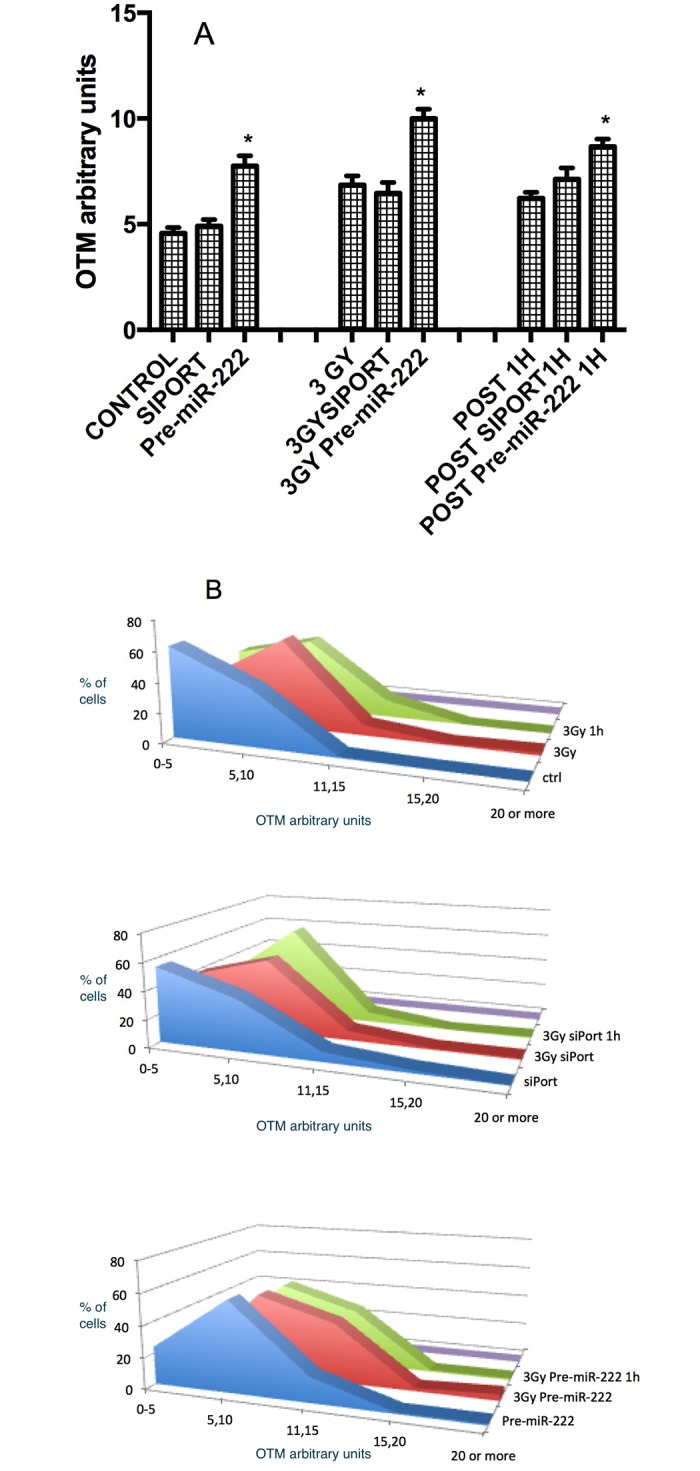
DNA damage analysis. **(A)** DNA DSBs in irradiated Balb/c 3T3 cells previously transfected with pre-miR-222 molecules at 5 min (3 Gy), 1 h post-exposure and evaluated with the neutral comet assay protocol. Results are presented in terms of Olive Tail Moment (OTM); dose of gamma radiation = 3 Gy, N = 2 slides/condition, 50 comets/slide, mean ± SE, two-tailed unpaired t-test, *p<0.05. **(B)** Distribution of DNA damage from pre-miR-222 cells treated with gamma-radiation (3Gy) and 1 h post-exposure, and analyzed by neutral comet assay.

We evaluated the presence of DNA DSBs in cells transfected with the pre-miR-222 and/or irradiated with gamma radiation at a dose of 3 Gy to challenge the HR mechanism response. Cells over-expressing miR-222 present DNA-DSB’s induction with respect to control (Fig 4A). In addition we observed a synergistic effect to induce DBA-DSB’s by γ-irradiation (3 Gy) (pre-miR-222/3 Gy) (10.01±0.44, control (3Gy) = 6.47±0.5) and 1 h post-irradiation (Fig 4A). The distribution of damage clearly shows DNA damage accumulation in cells with miR-222 over-expression (Fig 4B).

### Treatment with the precursor of miR-222 as an initiator stimulus

**Fig 5:**
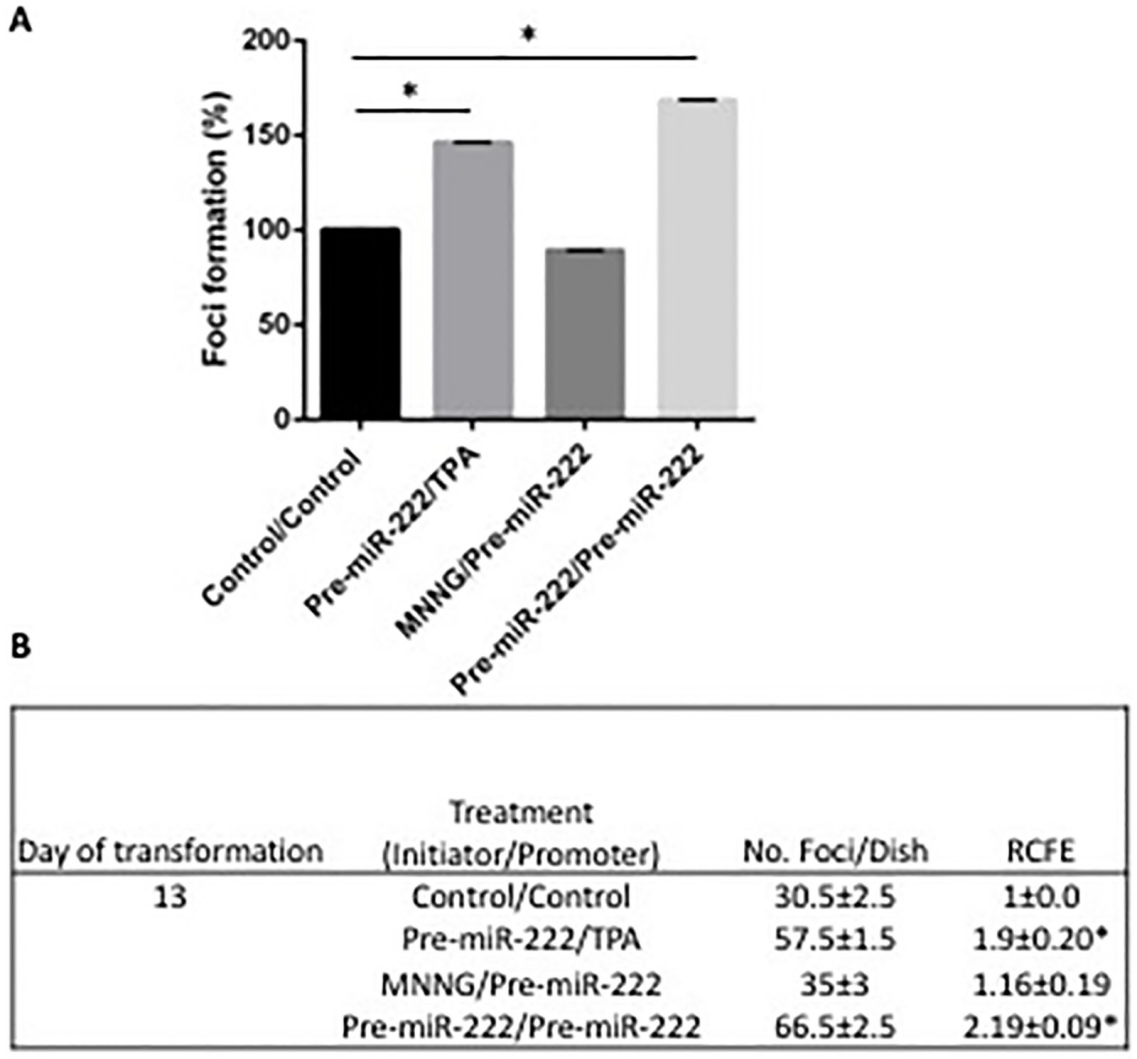
Percentage of *foci* generation (*Foci* %) in the Balb/c 3T3 cellular transformation assay. **(A)** Cellular transformation *foci* analysis on day 13 of the assay from cultures treated with the miR-222 precursor molecule as initiator stimulus and the known TPA promoter (pre-miR-222/TPA), with the known initiator MNNG and the miR-222 precursor molecule as promoter stimulus (MNNG/pre-miR-222) or with the miR-222 precursor molecule as initiator and promoter stimulus (pre-miR-222/pre-miR-222), compared to the control condition (Control/Control). (RCFEs were divided over the Control/Control condition of the respective day and multiplied by 100) N = 2, mean ± SE, one-way ANOVA *F* (5,6) = 49043 *p<0.0001, Tukey post-hoc comparisons of the groups 95% CI. **(B)** Relative colony formation efficiency (RCFE) of Balb/c 3T3 cells of day 13, for cells treated with the pre-miR-222 molecule as an initiator and/or promoter stimulus. N = 2, mean±SE, two-tailed un paired t-test, *p<0.05.

Finally, we evaluated the role of Rad51c regulation through miR-222 in the Balb/c 3T3 morphological transformation assay by transfection of the pre-miR-222 molecule and following the cultures until transformation. We observed in Fig 5 that percentage of *foci* formation in all conditions where pre-miR-222 was used as initiator stimuli, transformation appears on day 13 of the assay (Fig 5A).

Then, we calculated the RCFE (No. *foci*/dish of each experimental condition/No. *foci* per dish of the control of day 13) (Fig 5B). Employing pre-miRNA-222 as an initiator combined with a known promoter (TPA) or with pre-miRNA-222 also as promoter stimuli increased the RCFE significantly (1.9±0.2 and 2.19±0.09, respectively).

## Discussion

The cell viability of the Balb/c 3T3 cultures during the initiation stage of the transformation assay was not affected by transfection with the pre-miRNA-222 (Table 3), indicating that the results presented in this manuscript were not they owe to the cytotoxic effects.

We demonstrated the inhibition of Rad51c directly through the complementarity of its 3´UTR with the miR-222 sequence using a luciferase activity reporter assay “Fig 2”, confirming our bioinformatics prediction and validating a novel target for this small RNA.

When we manipulate the expression of the miRNA without compromising the cell viability (Table 3), we can appreciate the importance of miR-222 expression, specifically; a significant decrease in Rad51c gene and protein expression “Fig 3A and 3B”, respectively were observed when we induced its expression with pre-miR-222 transfection (Table 3). At this moment, it is important to note that the presence of miR-222 variants called isomirs have been reported recently [55,56]. These variants could have different implications in the microRNA function depending of their size, principally at the 3’ end. The presence of isomirs has been reported in almost all microRNAs, these could be longer or shorter than consensus length of the microRNAs. In the case of miR-222 it has been reported that the longest variants of about 23 to 24 nucleotides are present in almost all organs at concentrations almost similar to the canonical sequences of 21 base pairs. It has also been reported that long sequences induce apoptosis and short sequences are more related to cell survival. In our study because of the strategy used, we do not know with certainty if the cellular machinery for the production of microRNAs is generating both long or canonical sequences of the microRNA, although the designed premiR was the one corresponding to the sequence of 21 base pairs, however, in our transfected cultures do not appreciate an increase in apoptosis as Yu et al.,[55] found, so we consider that miR-222 overexpressed in our cultures is the canonical sequence of 21 base pairs.

After confirming the miR-222 up-regulation and Rad51c down-regulation, we tested for DSBs DNA repair in Balb/c 3T3 under those conditions by inducing DSBs with gamma radiation and testing its presence or absence at 5 min, 1 h after the challenge “Fig 4”. We observed an increase in DNA damage in cells over-expressing the miRNA alone, indicating that miR-222 plays a determinant role during the initiation of the transformation process through Rad51c inhibition and the consequent HR repair inhibition. This inhibition may lead to the characteristic damage accumulation and genomic instability of this stage of the process [1,5,9,10]. To confirm the important role that HR plays in the state of cellular transformation initiation, we inhibited RAD51, a central HR protein [56] with the chemical inhibitor RI-1, we observed greater DNA damage to the cells treated with doxorubicin when Rad51 was inhibited “S1A Fig”. Also in these cells treated with doxorubicin, we observed a greater number of cells with ATM and λH2AX phosphorylated indicating greater DNA damage “S1B Fig”.

This effect was also detected in the cells 5 min and 24 h post-irradiation, confirming the role of miR-222 as demonstrated by the exacerbation on DNA damage.

Finally, we needed to elucidate whether the effects of miR-222 are relevant to the cellular transformation endpoint assessed by morphological changes and *foci* formation in Balb/c 3T3 model [39,48,57], we observed that the use of the pre-miR-222 as an initiator (combined with the known promoter TPA [56] accelerates transformation and generates *foci* on day 13 “Fig 5”. Furthermore, pre-miR-222 also produce more *foci* number when it is used as a initiator and promoter stimulus, but not when is used only as a promoter stimulus (along with the known initiator agent MNNG [58,59]), confirming the cellular consequences of elevated levels of this miRNA. We also run the transformation assay and see that antimiR-222 when was used as initiator stimulus and pre-miR-222 as promoter, cell transformation was inhibited and when was used as a promoter stimulus and pre-miR-222 as initiator, the transformation was reduced significantly (data not show). When we performed the Two stage Balb/c 3T3 cell assay in the cells with RAD51 inhibited, we observed an increase in the amount of foci, similar to that found when RAD51c was inhibited with the overexpression of miR-222 “S1C Fig”. These results indicate that the participation of HR is essential to avoid cell transformation.

Our results indicate that the up-regulation of miR-222 plays an important role as an initiator of the carcinogenesis but not in the promotion process. Thus, miR-222 plays an essential role early in the process or carcinogenesis inhibiting HR and is not merely a marker of various established cancers [40–43].

## Conclusions

We were able to demonstrate that miR-222 over-expression has serious repercussions; it impairs homologous recombination-mediated DNA DSB repair as well as induction of morphological transformation in the Balb/c 3T3 *in vitro* model. Our results suggest that an increase in miR-222-mediated Rad51c inhibition contributed to the loss of genomic stability, initiating the carcinogenesis process.

## Supporting information

S1 Fig A. Percentage of DNA Damage in cells pre-treated with a RAD51-inhibitor RI-, after doxorubicin treatment. DNA damage is expressed as a Tail DNA, Olive tail moment (OTM) and Tail length. mean ± SE, two-tailed unpaired t-test, * p<0.5, *** p< 0.001. B. Percentage of cells treated with Doxorubicin and RI-1 with ATM and λH2AX phosphorylated. mean ± SE, two-tailed unpaired t-test, * p<0.5, ** p<0.01. C. Number of Cellular transformation *foci* on day 13 of the assay from cultures treated with the RI-1 as initiator stimulus and the known TPA promoter (RI-1/TPA), with the known initiator MNNG and the RI-1 as promoter stimulus (MNNG/RI-1) or with the RI-1 as initiator and promoter stimulus (RI-1/RI-1), compared to the control condition (Control). mean ± SE, one-way ANOVA * p<0.05 **p<0.01.
(TIFF)

S1 File Compendium of data used to generate the manuscript. (TBZ2)
