# Supplementary figures and images for: Post-transcriptional regulation of Rad51c by miR-222 contributes cellular transformation
Source: PLoS One. 2020 Jan 10;15(1):e0221681. doi: 10.1371/journal.pone.0221681 (PMC6953820; doi:10.1371/journal.pone.0221681)

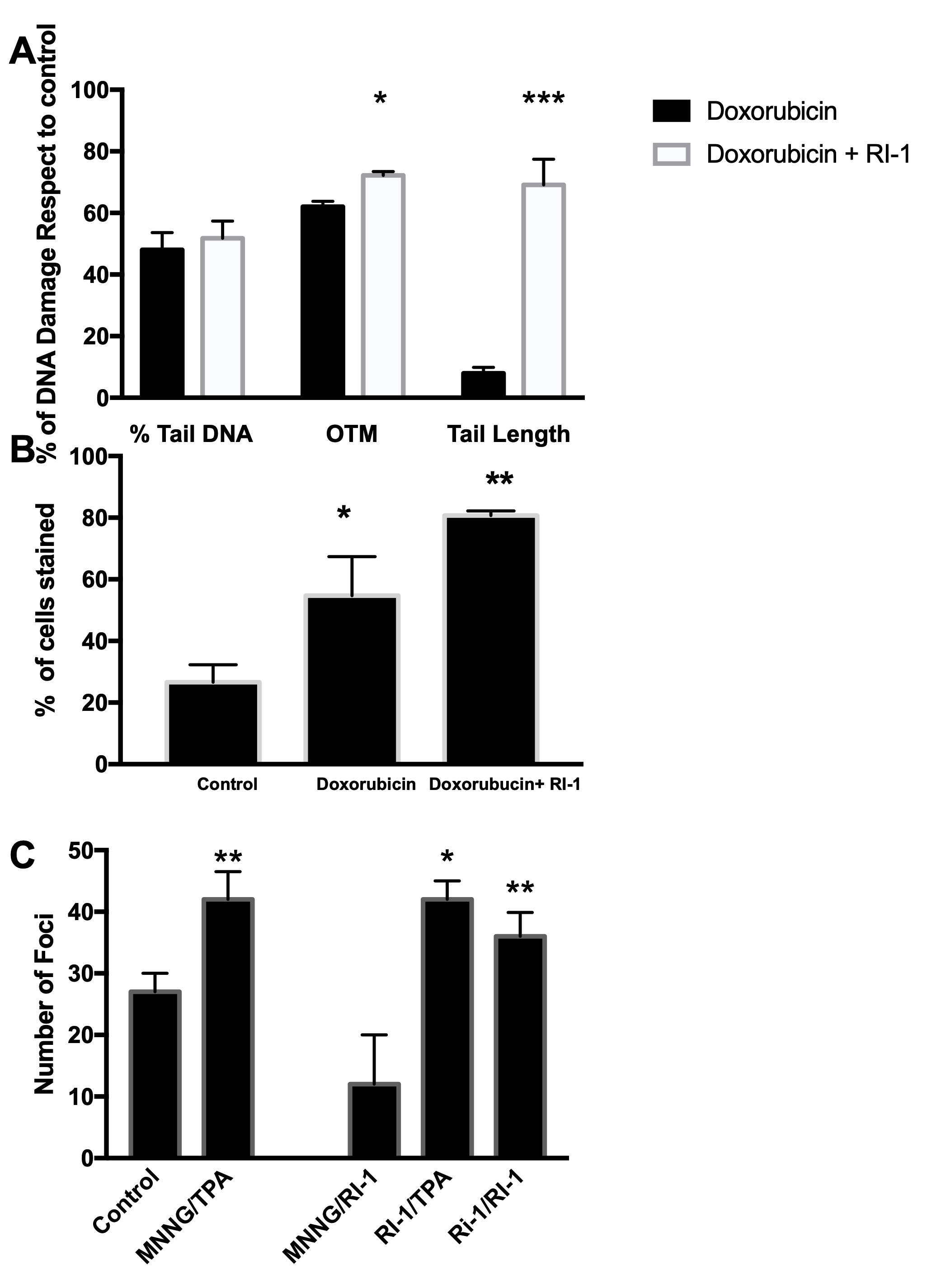

Supplement: S1 Fig — A. Percentage of DNA Damage in cells pre-treated with a RAD51-inhibitor RI-, after doxorubicin treatment. DNA damage is expressed as a Tail DNA, Olive tail moment (OTM) and Tail length. mean ± SE, two-tailed unpaired t-test, * p<0.5, *** p< 0.001. B. Percentage of cells treated with Doxorubicin and RI-1 with ATM and λH2AX phosphorylated. mean ± SE, two-tailed unpaired t-test, * p<0.5, ** p<0.01. C. Number of Cellular transformation foci on day 13 of the assay from cultures treated with the RI-1 as initiator stimulus and the known TPA promoter (RI-1/TPA), with the known initiator MNNG and the RI-1 as promoter stimulus (MNNG/RI-1) or with the RI-1 as initiator and promoter stimulus (RI-1/RI-1), compared to the control condition (Control). mean ± SE, one-way ANOVA * p<0.05 **p<0.01. (TIFF) [file pone.0221681.s001.tiff]
